# Are atrial human pluripotent stem cell-derived cardiomyocytes ready to identify drugs that beat atrial fibrillation?

**DOI:** 10.1038/s41467-021-21949-z

**Published:** 2021-03-19

**Authors:** Torsten Christ, Marc D. Lemoine, Thomas Eschenhagen

**Affiliations:** 1 Institute of Experimental Pharmacology and Toxicology, Cardiovascular Research Center, University Medical Center Hamburg-Eppendorf, Hamburg, Germany; 2 DZHK (German Centre for Cardiovascular Research), partner site Hamburg/Kiel/Lübeck, Hamburg, Germany; 3 Department of Cardiology-, University Heart and Vascular Center, Hamburg, Germany

**Keywords:** Cardiology, Medical research

**Arising from** Goldfracht et al. *Nature Communications* 10.1038/s41467-019-13868-x (2020)

A key issue in the development of atrial-selective antiarrhythmic drugs is the limited access to human heart tissue. Goldfracht et al. have used atrial- and ventricular-differentiated human embryonic stem cell‐derived cardiomyocytes (hESC-CMs) to dissect chamber-selective actions of clinically relevant antiarrhythmic drugs^1^. This is plausible, but we want to point to remaining differences between the electrophysiological properties the atrial hESC-CMs presented in the study and adult human atrial cardiomyocytes. We believe that further refinement and in-depth comparison of atrial- and ventricular-differentiated hESC-CM with adult human cardiomyocytes and tissue is warranted before these models can be safely used for the development of atrial-selective antiarrhythmics.

There is an unmet need to find new drugs to stop atrial fibrillation (AF) without inducing ventricular arrhythmias. However, access to atrial and even more to ventricular human tissue is limited, both of which could be principally solved by the widespread availability of human induced pluripotent stem cell‐derived cardiomyocytes (hiPSC-CM) or hESC-CMs, which are limited to some countries due to ethical requirements. Indeed, protocols to generate atrial hiPSC-CM or hESC-CM have been established^2,3^. However, differences in antiarrhythmic drug response between atrial and ventricular EHTs have only scarcely been investigated^3,4^. Therefore, the study by Goldfracht et al. using established antiarrhythmic drugs is very welcomed^1^. The authors used the early method of ring-shaped EHTs^5^, which allowed the induction of macro reentry arrhythmia, visualized by voltage sensitive dyes, and the exploration of activation maps and drug effects.

Goldfracht et al. used vernakalant, a compound approved in the EU since 2010 as an antiarrhythmic drug in AF. The authors used this multichannel blocker because it blocks, amongst others, two ion currents selectively present in the atria and not in the ventricle: the ultrarapidly activating potassium outward rectifier current (*I*_Kur_) and the acetylcholine-activated potassium inward rectifier current (*I*_K,ACh_)^6^. The underlying hypothesis was that effects of vernakalant in atrial, but not ventricular EHTs are indicative of a truly atrial phenotype. Indeed, vernakalant (30 µM) increased action potential duration at 90% percent of repolarization (APD_90_) in atrial EHTs by about 100% (~200 ms), while it was reported ineffective in ventricular EHTs.

Of note, however, the large effect of vernakalant on APD_90_ in atrial EHTs is in stark contrast to results obtained in human atrial tissue, where the same concentration of vernakalant did not prolong APD_90_ at all^6^. Inefficacy of *I*_Kur_ block to prolong APD_90_ in human atrium is a common finding and is explained by indirect activation of the rapid component of the delayed rectifier potassium current (*I*_Kr_)^7^. Thus, one has to consider block of another potassium channel to underlie the marked prolongation of APD_90_ in atrial EHT upon vernakalant. Contribution of *I*_K,ACh_ to repolarization normally requires G protein-coupled inwardly rectifying potassium channels to be open, e.g., by stimulation of muscarinic or adenosine receptors. However, agonists should be absent under in vitro conditions. Thus, other scenarios have to be considered.

Atrial hiPSC-CMs or hESC-CMs could differ from human atrium with respect to repolarization reserve (similar to what we have reported for ventricular EHTs^8^), enabling block of *I*_Kur_ to prolong APD_90_.

A high baseline activity of *I*_K,ACh_ could result either from acetylcholine or adenosine present in these cultures or from constitutively active *I*_K,ACh_, as reported in human AF^9^.

Alternatively, vernakalant could have induced APD_90_ prolongation in atrial hESC-CM by blocking ion channels normally absent from adult CM, but present in immature hESC/hiPSC-CM, an example for this phenomenon was reported recently^10^.

In contrast to the unusual effect in atrial EHTs, the reported absence of effects of vernakalant on repolarization in ventricular EHTs appears, at first sight, to match the reported vernakalant effects on human ventricular tissue^6^. However, the statistical approach used by Goldfracht et al.^1^ may be suboptimal to safely exclude effects of vernakalant. Given the clinical relevance of prolongation of repolarization, the United States Food and Drug Administration (FDA) requires the analysis of drug effects on ventricular repolarization to be done in a pairwise manner, i.e., comparing single values in the presence and absence of the test compound to detect even small changes of repolarization^11^. The data presented in Figure 3e of Goldfracht et al.^1^ (which have been evaluated in an unpaired manner) in fact indicate that APD_90_ in the vernakalant-treated group of cells was ~40 ms longer than in the vehicle group. A randomized phase 3 clinical trials showed that vernakalant prolongs QT_c_ in patients by ~20 ms^12^. The effect size was confirmed in several other trials. The prolongation may be small, but could be relevant, and underlie the ongoing concern about the safety of vernakalant. In fact, FDA again rejected to approve vernakalant in the US^13^. We are afraid that the methodology used by Goldfracht has weakened the power to confirm established repolarization delay by vernakalant in ventricular EHT.

The authors also measured slowing of conduction as a surrogate parameter of sodium channel block. Of note, the basal upstroke velocities of ventricular and atrial EHTs action potentials in this study (12 and 7 V/s, Fig 1e) were very low in comparison to the literature (207 and 98 V/s)^3^, and this abnormality is paralleled by depolarized take-off potentials of >−60 mV (Fig 1d) (normal −70 and −76 mV)³. This range of upstroke velocities observed is typical of Ca^2+^-dependent action potentials in which Ca^2+^-channel provide most of the depolarizing current during the upstroke. This indicates immaturity of the hESC-CM and limits the transferability of the in vitro results with a sodium current blocker. The sodium channel blocker lidocaine (100 µM) slowed down conduction in ventricular but not in atrial EHT^1^. The authors correctly point out that lidocaine is unable to convert AF in patients. However, we do not agree with the conclusion that the absence of lidocaine-induced conduction slowing in atrial EHT can be taken as an indication of an atrial phenotype. Even moderate concentrations of lidocaine (≤10 µM) slowed conduction both in canine ventricle and atria^14^. The same concentration of lidocaine used by Goldfracht et al. (100 µM) decreased both maximum upstroke velocity of action potentials and conduction velocity in atrial tissue from rabbits and guinea-pigs^15^. No in vitro data on human tissue are available, but the published data clearly argue against the usefulness of lidocaine to characterize an atrial drug response pattern.

Taken together, we would like to raise a word of caution when using atrial hiPSC-CMs or hESC-CMs as a model to dissect human atrial and ventricular electrophysiology. It remains unclear whether the unexpected large prolongation of APD_90_ by vernakalant as well as the lack of lidocaine to slow conduction is a peculiarity of this individual cell line, the culture conditions or the retinoic acid-based protocol to induce atrial differentiation. Undisputedly, the depolarized MDP and low upstroke velocity in both atrial and in ventricular cells indicate an immature, embryonic like CMs phenotype with its specific physiology and pharmacology. As a result of immaturity, disease modeling using hiPSC-CMs or hESC-CMs has been considered “of limited value if the cells are not adequately characterized with respect to their electrophysiology, contractility, kinetics, etc”^16^. We agree with Goldfracht et al. regarding the huge potential of atrial hiPSC-CMs or hESC-CMs. However, further refinement of atrial differentiation appears warranted to develop the full potential of these new models for cardiac electrophysiology and drug development.

## Data Availability

No data sets were generated or analyzed during the current study.
